# Study of the uncertainty in the determination of the absorbed dose to water during external beam radiotherapy calibration

**DOI:** 10.1120/jacmp.v9i1.2676

**Published:** 2008-01-22

**Authors:** Pablo Castro, Feliciano García‐Vicente, Cristina Mínguez, Alejandro Floriano, David Sevillano, Leopoldo Pérez, Juan J. Torres

**Affiliations:** ^1^ Servicio de Oncología Radioterápica, Departamento de Radiofísica Hospital Universitario “La Princesa” Madrid Spain

**Keywords:** Uncertainty in dosimetry, beam calibration, absorbed dose

## Abstract

To achieve a good clinical outcome in radiotherapy treatment, a certain accuracy in the dose delivered to the patient is required. Therefore, it is necessary to keep the uncertainty in each of the steps of the process inside some acceptable values, which implies as low a global uncertainty as possible. The work reported here focused on the uncertainty evaluation of absorbed dose to water in the routine calibration for clinical beams in the range of energies used in external‐beam radiotherapy. With this aim, we considered various uncertainty components (corrected electrometer reading, calibration factor, beam quality correction factor, and reference conditions) associated with beam calibration. Results show a typical uncertainty in the determination of absorbed dose to water during beam calibration of approximately 1.3% for photon beams and 1.5% for electron beams (k=1 in both cases) when the $N_{D,w}$ formalism is used and $k_{Q,Q_{0}}$ is calculated theoretically. These values may vary depending on the uncertainty provided by the standards laboratory for calibration factor, which is shown in the work. For primary standards based on clinical linear accelerator beam energies, the uncertainty in this step of the process could be placed close to 1.0%. We also discuss the possibility of an uncertainty reduction with the adoption of the absorbed dose to water formalism as compared with the air kerma formalism.

PACS numbers: 87.53.Dq, 87.53.Hv

## I. INTRODUCTION

The development of new techniques in external radiotherapy has led to an increase in the complexity of the procedures used. Treatment delivery to the patient therefore involves many steps, parameters, and factors. As a result, more complex quality controls are required during the radiotherapy process to ensure that each step has as low an uncertainty as possible. On the other hand, developments in the technology of detectors, in dose‐calculation algorithms, and in the stability of accelerators can lead to a decrease in that uncertainty.

The reason for trying to minimize uncertainty is the need, at a clinical level, to deliver dose to the patient within a determined accuracy. That clinical requirement is based on dose–response curves for tumor control probabilities (TCPs) and for normal‐tissue complication probabilities (NTCPs) alike—TCPs and NTCPs being directly related to the deviation between the prescribed dose and the actual absorbed dose. The uncertainty in the dose delivered to the target volume involves higher or lower variations on TCP and NTCP parameters depending on the slope of the curve. In general, accuracy requirements are defined for the most critical response curves—that is to say, the curves with a steeper gradient.

Thus, in an effort to understand the desirable accuracy of the therapeutics process, Boyer and Schultheiss^(1)^ concluded that an accuracy improvement of 1% is associated with a 2% increase in early‐stage tumor control. Mijnheer et al.^(2)^ presented less‐critical values for bladder treatments: an increase of 1% in tumor control for every 1% reduction in uncertainty.

From clinical data, several authors and organizations have established recommendations for the accuracy required in radiotherapy treatment. For example, through a TCP data recompilation, report 24 of the International Commission on Radiation Units and Measurements^(3)^ claims the need for 5% accuracy (and in some cases even 2%), although a coverage factor is not set. Frequently, a value of 1.5 or 2 is assigned, meaning 86.6% and 95.5% coverage probability respectively. These values have been corroborated by several studies: Brahme et al.^(4)^ concluded that, for some kind of tumors (such as larynx), the uncertainty should be below 3.3%. Mijnheer et al.,^(2)^ considering the effect of dose variations over both TCP and NTCP for various kinds of tumors, proposed 3.5%, and although that particular level means that higher values are acceptable for most cases, in certain tumors the aim should be toward lower values.

Such accuracy levels are, however, not currently achievable in most treatments (dose escalation in three‐dimensional conformal radiotherapy, radiosurgery, and so on). In fact, more realistic recommendations, about 5%, are made by the same authors in another publication,^(5)^ considering the additional uncertainty from dose‐calculation algorithms.

All of that background points to the importance of evaluating the uncertainties introduced at every step associated with the radiotherapy process. Given the current state of the art, global process uncertainty should be approximately 6%.^(6)^

The present work focuses on the evaluation and quantification of the uncertainty associated with the determination of absorbed dose during calibration of photon and electron beams. Recent estimates have yielded a typical uncertainty value in this step of the process of between 1.4% and 2.1%^(^ ^6^ ^–^ ^8^ ^)^—the largest contribution to the uncertainty being associated with the factor $k_{Q,Q_{0}}$.^(^ ^7^ ^,^ ^8^ ^)^

The calibration protocol used in the present work is TRS‐398^(8)^ provided by the International Atomic Energy Agency (IAEA). Adoption of that protocol, which is based on absorbed dose to water, implies several improvements over protocols based on air kerma. *A priori*, one of these improvements is a reduction in the uncertainty during determination of the absorbed dose to water. However, a recent publication,^(7)^ which is also analyzed in the present work, has questioned this argument.

The evaluation of uncertainties as presented here follows the method described in “Expression of the uncertainty of measurement in calibration” (EAL‐R2)^(9)^ in its Spanish version, CEA.ENAC‐LC/02,^(10)^ supplied by Entidad Nacional de Acreditación, which follows recommendations proposed by the International Organization for Standardization.^(11)^

## II. MATERIALS AND METHODS

The calibration procedure for external‐beam radiotherapy calibration is TRS‐398.^(8)^ It is based on measurements of absorbed dose to water under reference conditions. An ionization chamber set in a water phantom is used to take the measurements. The reference conditions change with the type of radiation and the beam quality, and these conditions are described in the protocol.

Our study was carried out using two beam modalities in the energy range common to radiotherapy: photons with nominal energies of 6 MV and 25 MV, and electrons with nominal energies of 6, 9, 12, 15, 18, and 21 MeV produced by the linear accelerators Saturn 40 and Saturn 43 (General Electric Medical Systems, Buc, France).

The ionization chambers used to obtain absolute measurements of dose must be traced to primary standards. Primary standards use a measurement method of primary character to determine the absorbed dose to water. Only three methods have the primary character necessary to determine the absorbed dose to water: calorimetry, chemical dosimetry, and ionization dosimetry. The owners of the primary standards are called primary standard dosimetry laboratories (PSDLs). Most of the PSDLs operate a C60o gamma‐ray beam to disseminate the absorbed dose to water to the secondary standards of the secondary standard dosimetry laboratories (SSDLs). The SSDLs calibrate the reference instruments of users.

We used a PTW Farmer cylindrical chamber (PTW, Freiburg, Germany) with a PTW MP3TANDEM electrometer to calibrate photon beams and a PTW Markus parallel‐plate chamber with the PTW MP3TANDEM electrometer to calibrate electron beams. The ionization chambers and the electrometer were separately calibrated for absorbed dose to water in C60o beam quality by the manufacturer, which is a SSDL and is traceable to the PTB (Physikalisch‐Technische‐Bundesanstalt) PSDL in Germany. The latter laboratory used chemical dosimetry as the primary measuring method. The primary standard was the Fricke dosimeter. (The present work was carried out before that standard was replaced by a water calorimeter.)

In addition, we used another assembly consisting of a NE 2571 cylindrical chamber and a NE 2570–1 electrometer to reflect the influence of the laboratory of calibration on the uncertainty estimation. This assembly was calibrated by the Ciemat (Centro de Investigaciones Energéticas, Medioambientales y Tecnológicas), a SSDL traceable to the Bureau International des Poids et Measures (BIPM). Both laboratories use ionization dosimetry as the measuring method.

We also used these additional measurement devices:

- a PTW MP3 beam analyzer,
- a 2039.70492 Lufft analog precision barometer (Lufft, Fellbach, Germany), and
- a Unitest Beha 93450 digital thermometer (Beha‐Amprobe, Glottertal, Germany).

We applied the IAEA TRS‐398 formalism, in which determination of the absorbed dose to water in reference conditions for a beam of quality *Q* in the chamber cavity is formulated. The final expression for determination of the absorbed dose (to be used as the initial formula for our uncertainty calculation) is

(1) $$\begin{array}{l} D_{w,Q}=M_{Q}\cdot k_{TP}\cdot {\left(k_{h}\right)}_{Q}\cdot {\left(k_{elec}\right)}_{Q}\cdot {\left(k_{pol}\right)}_{Q}\cdot {\left(k_{S}\right)}_{Q}\cdot \frac{N_{D,w,Q_{0}}}{{\left(k_{pol}\right)}_{Q_{0}}\cdot {\left(k_{S}\right)}_{Q_{0}}}\cdot k_{Q,Q_{0}}\\ \;=M_{Q}^{*}\cdot N_{D,w,Q_{0}}\cdot k_{Q,Q_{0}}, \end{array}$$

with the meaning of the various terms being given in TRS‐398. The standards laboratory does not explicitly correct for the effects of polarity or saturation. For suitability in calculation, those two factors, polarity and saturation, have been included in the corrected reading $M_{Q}^{*}$.

Assuming no correlation between the components, the expression of the relative combined standard uncertainty yields

(2) $$\frac{u_{c}^{\;2}\left(D_{w,Q}\right)}{D_{w,Q}^{\;2}}={\left(\frac{1}{M_{Q}^{*}}\right)}^{2}\cdot u^{2}(M_{Q}^{*})+{\left(\frac{1}{N_{D,w,Q_{0}}}\right)}^{2}\cdot u^{2}(N_{D,w,Q_{0}})+{\left(\frac{1}{k_{Q,Q_{0}}}\right)}^{2}\cdot u^{2}(k_{Q,Q_{0}}).$$

Because the expression of the absorbed dose contains only products and quotients, the uncertainty will be the quadratic sum of the uncertainties associated with each component. Furthermore, an additional uncertainty attributable to reference conditions has to be added. Thus, all of these components must be analyzed and estimated in agreement with the 1997 EAL‐R2 document^(9)^ to obtain the standard uncertainty in the determination of the absorbed dose during the beam calibration process.

The standard uncertainties are classified into A and B types. This classification indicates the two different ways of evaluating uncertainty components. Both types are based on probability distributions, but a type A standard uncertainty is obtained from a series of repeated observations, and a type B standard uncertainty is evaluated by methods other than a statistical analysis of a series of observations.

As is mentioned in the 1997 EAL‐R2 document,^(9)^ the variation limits of an analyzed component could be well known, but their distribution unknown. In these cases (common in this work), a rectangular (also known as “uniform”) distribution that gives a constant value inside the interval and a null value outside it is considered. If the maximum variation limits are given by −a and +a, then the type B standard uncertainty is given by $u_{B}=a/3^{1/2}$. On the other hand, and to avoid misunderstandings, a coverage factor of k=1 is taken for every uncertainty value, corresponding to a confidence limit of 68.3%.

## III. RESULTS

### A. Corrected reading $M_{\;Q}^{*}$

The components that we took into account were

- charge reading,
- monitor chamber,
- pressure,
- temperature,
- relative humidity,
- electrometer calibration,
- polarity, and
- saturation.

The values analyzed in the subsections that follow correspond to the uncertainty associated with the PTW M30013 chamber for photon beams and the Markus chamber for electron beams. Later, we present the data for the NE 2571 measuring photons.

#### A. 1 Charge reading

##### A. 1.1 Values

(3) $$M_{Q}=M_{\text{Display}}-M_{\text{Leakage}}≅M_{\text{Display}}$$

The value of $M_{\text{Leakage}}$ can be determined by measuring the collected charge in the absence of radiation after and before the irradiation. That reading can then be used to verify that the reading from the electrometer is $M_{\text{Display}}\geq 10^{3}\;M_{\text{Leakage}}$, as recommended in TRS‐398, and so on, accomplishing condition (3).

##### A. 1.2 Uncertainties

(4) $$u^{2}(M_{Q})=u^{2}(M_{\text{Display}})+u^{2}(M_{\text{Leakage}})$$

No correlation between $M_{\text{Display}}$ and $M_{\text{Leakage}}$ is assumed in the foregoing expression, even though the same electrometer is used to measure both. This approach is acceptable because the uncertainty associated with leakage is very small, as is the measure itself.

The uncertainties of the following components were analyzed in the measuring assembly for measurement of charge:

- **Reproducibility**. Reproducibility is a standard type A uncertainty that can be deduced from a series of repeated measurements under the same conditions. For both measuring assemblies (cylindrical and plane‐parallel chambers with electrometer), we made a series of repeated measurements in a C60o gamma‐ray machine. By using this approach, we avoided fluctuations of the accelerator between irradiations. Neither the rate nor the type of radiation is the same as in the accelerator. However, the aim was to estimate the reproducibility of the measurement system, regardless of the measurement conditions. The uncertainty was estimated at 0.03%, based on a k=1 standard deviation of the mean.
- **Display resolution**. From the smallest unit of measurement that can be indicated by the electrometer, and assuming a rectangular distribution, we obtained an uncertainty value of 0.01% for both the cylindrical and the plane‐parallel chamber. These values are useful for the whole range of energies considered.
- **Electrometer linearity**. Linearity was evaluated using a S90r check source, measuring charge at various times (dose is directly proportional to time and so to the charge reading). Maximum deviation of the linearity was found to be 0.06%. In consequence, considering a rectangular distribution, the uncertainty associated with this component is 0.03%.
- **Zero of the electrometer**. In actuality, $M_{\text{Display}}=M-m$, in which *m* is 0 or slightly different. And an uncertainty will be associated with the electrometer resolution. A value equal to the uncertainty for the display resolution (0.01%) has been considered for both the cylindrical and the plane‐parallel chambers.
- **Long‐term stability**. The cylindrical chamber shows some variation over time, as indicated by evaluations of its calibration as measured with the S90r check source every month over a period of 2 years. The standard deviation related to these measurements was 0.29%.
  We used the difference between two calibration factors (in two consecutive calibrations) to evaluate the variation over time demonstrated by the plane‐parallel chamber. A rectangular distribution was centered in one of the values. The uncertainty was placed at 0.48%. The lack of data makes this value the best estimation of the chamber response variation over time.
- **Leakage**. A complex evaluation is required for the leakage component. As an approximation, this contribution was overestimated as being equal to the reading resolution, although the contribution of this component is smaller. Using this procedure, the uncertainty attributable to leakage will fall with high probability inside the real value.

Table 1 summarizes the uncertainty values of the various components associated with the electrometer reading. The combined uncertainty is obtained by determining the square root of the squares of the relative component standard uncertainties. The component associated with long‐term stability makes the most important contribution to the uncertainty in the reading.

**Table 1:** Estimated relative standard uncertainty of the electrometer reading and of its components, for two chambers types: a cylindrical PTW 30013 (PTW, Freiburg, Germany) and a plane‐parallel Markus chamber (PTW)

Component	Photons, M30013			Electrons, Markus		
	Uncertainty type A (%)		Uncertainty type B (%)	Uncertainty type A (%)		Uncertainty type B (%)
Reproducibility	0.03			0.03		
Resolution			0.01			0.01
Linearity			0.03			0.03
Zero			0.01			0.01
Long‐term stability			0.29			0.48
Leakage			0.01			0.01
**Combined uncertainty** (k=1)		**0.29**			**0.48**	

#### A. 2 Beam monitor

##### A.2.1 Values

Selected monitor units state the reading time, which is why this component needs to be analyzed.

##### A.2.2 Uncertainties

The common practice during routine beam calibration is to use a unique shot, always with the same monitor units (200 MUs), to evaluate dose. Possible variations in machine output from the calibration date should be studied in another step of the process, when treatment delivery to the patient is carried out. Consequently, the only component to bear in mind in selecting MUs is the display resolution (±0.5 MU). Considering a rectangular distribution, an uncertainty of 0.14% is obtained—$u(\%)=[0.5/(200•3^{1/2})]•100$.

#### A.3 Correction factor for pressure

##### A.3.1 Values

The factor is $P_{0}/P$, where $P_{0}=101.3$ kPa.

##### A.3.2 Uncertainties

The following uncertainty components are assumed:

- **Discrimination threshold**. The minimum scale division is 0.5 mb. The discrimination threshold is a subjective parameter that depends on the visual acuity of the observer, as well as on the separation and the width of the marks in the scale division. It has been stated in thirds of the minimum scale division. Therefore, considering a rectangular distribution, an uncertainty value of 0.096 mb is obtained.
- **Calibration certificate**. According to the calibration certificate, the uncertainty associated with the range of the values registered in measurement conditions is 0.2 mb (920 ‐ 980 mb is the approximate maximum pressure variation in Madrid).
- **Correction to be applied**. For the same range and according to the calibration certificate, a correction of 0.06 mb should be applied to our readings. Because the barometer resolution is 0.5 mb, the foregoing correction cannot be applied. Thus, despite not being applied, 0.06 mb is associated with the uncertainty value.
- **Long‐term drift**. The difference between two consecutive calibrations provides an idea of the value of this component. Assuming a rectangular distribution centered on an extreme, an uncertainty value of 0.8 mb can be deduced for this term.

Table 2 summarizes the values of the various components analyzed for the correction factor for pressure and the combined uncertainty. The uncertainty components shown in the table are relative to a typical measuring pressure of 935 mb. If other pressure values were maintained during calibration, changes in the uncertainty values would not be significant.

**Table 2:** Estimated relative standard uncertainty of the correction factor for pressure and of its components^a^

Component	Uncertainty (%)
Discrimination threshold	<0.01
Calibration certificate	0.02
Non‐applied correction	<0.01
Long‐term drift	0.09
**Combined uncertainty** (k=1)	**0.09**

a All values arise from type B uncertainty analysis.

#### A.4 Correction factor for temperature

##### A.4.1 Values

The factor is $\left(273.2+T)/(273.2+T_{0}\right)$, where $T_{0}=20{\;}^{ΰ}C$.

##### A.4.2 Uncertainties

We considered these components:

- **Resolution**. The device resolution is 0.1 °C. Assuming a rectangular distribution, we estimated an uncertainty value of 0.06 °C.
- **Calibration certificate**. According to the calibration certificate, the uncertainty associated with the range of values that includes typical calibration temperatures (15–25 °C) is 0.29 °C.
- **Correction to be applied**. The calibration certificate states that a correction of +0.3 °C must be added to the reading for the typical range of temperatures during measurements. However, we did not use a calibration factor, and so the amount of the correction (0.3 °C) is included in the uncertainty.
- **Long term drift**. The deviation between two consecutive calibration factors provides an approximate value for this component. Assuming a rectangular distribution centered on an extreme, the value of this deviation can be deduced to be 0.06 °C.

On the other hand, in a recent report, Das and Zhu^(12)^ showed that chamber response is affected by the temperature from the water phantom, such that the materials that constitute the chamber may expand or contract. For model PTW 30006, which is equivalent to model PTW 30013, the maximum correction to apply has a value of about 0.19% for the temperature range at which measurements are carried out (15 – 25 °C). The uncertainty is equal to the value of the non‐applied correction. Again, a rectangular distribution is associated. For plane‐parallel chambers, a similar uncertainty to that obtained for the cylindrical chamber is assumed, although the effect may be less, because of the chamber geometry itself.

Table 3 summarizes the relative uncertainties of the various components analyzed, in which the thermal effect is included. The components, except this last one, are relative to a typical thermometer reading of 21 °C. Variations on readings within the interval 15 – 25 °C have a negligible influence over the uncertainties shown.

**Table 3:** Estimated relative standard uncertainty of the correction factor for temperature and of its components^a^

Component	Uncertainty (%)
Resolution	0.02
Calibration certificate	0.10
Non‐applied correction	0.10
Long term drift	0.02
Thermal effect	0.11
**Combined uncertainty** (k=1)	**0.18**

a All values arise from type B uncertainty analysis. The uncertainty attributable to the thermal effect that can cause a change over the sensitive volume of the chamber has been included.

#### A.5 Humidity ${\left(k_{h}\right)}_{Q}$

##### A.5.1 Values

No corrections for this factor are needed if relative humidity ranges between 20% and 80% (a condition satisfied in our measurements) and whenever the calibration factor refers to a relative humidity of 50%. For our chambers, the SSDL established measurement conditions between 40% and 60% relative humidity, and so a value of 1.000 can be assigned to this factor.

##### A.5.2 Uncertainties

As Das and Zhu^(14)^ state, if no correction is made for this effect, a maximum error of 0.3% can be reached in the range of 0% ‐ 100% relative humidity. That value is considered more than enough to handle this non‐applied correction. A rectangular distribution can be assumed, being 0.3% the variation limit. Thus, the uncertainty associated with this component is calculated to be $0.17\%[u(\%)=0.3\%/3^{1/2}]$.

#### A.6 Electrometer calibration ${\left(k_{elec}\right)}_{Q}$

##### A.6.1 Values

An electrometer calibration correction factor is employed when the ionization chamber and the electrometer are calibrated separately. (If they are calibrated together, ${\left(k_{elec}\right)}_{Q}$ is not required.) The calibration certificate states that ${\left(k_{\text{elec}}\right)}_{Q}=1.000\pm 0.25\%$.

##### A.6.2 Uncertainties

The estimated standard uncertainty is 0.14%, assuming a rectangular distribution with the variation limits set at ±0.25%.

#### A.7 Polarity ${\left(k_{\text{pol}}\right)}_{Q}/{\left(k_{pol}\right)}_{Q_{0}}$

##### A.7.1 Values

(5) $$k_{\text{pol}}=(|M_{+}|+|M_{-}|)/2M_{+}$$

- **User**. The polarity factor was set experimentally by means of a series of measurements repeated under the same conditions, with only the polarity changing. A reference chamber was also used to minimize possible fluctuations in the output. Furthermore, to avoid instrument correlation, different electrometers were used. Maximum deviation on measurement was ensured to be less than 0.2% and 0.3% for photons and electrons respectively. In view of the various publications in which a significant dependence of this factor on energy was shown,^(^ ^13^ ^–^ ^17^ ^)^ the ${\left(k_{\text{pol}}\right)}_{Q}$ values were determined for all available energies for electron beams measured with plane‐parallel chambers.
- **Standards laboratory**. As has been reported, the polarity effect for photon beams and cylindrical chambers does not significantly depend on energy,^(18)^ and so the same value can be assumed both for C60o beam quality and our photon beam qualities (<0.1%). For the Markus chamber, values below 0.2% have been reported.^(19)^ However, a value of 1.000 is assigned to ${\left(k_{\text{pol}}\right)}_{Q0}$ for both chambers and then an uncertainty equal to this non‐applied correction is assigned (0.1% for the cylindrical chamber and 0.2% for the plane‐parallel chamber).

##### A.7.2 Uncertainties

- **User**. Type A uncertainties of the readings in both polarities are evaluated to establish the uncertainty associated with the polarity factor. By taking into account the machine reproducibility for measurements, the standard deviation is 0.1% and 0.15% for photons and electrons respectively. (A series of 7 samples was used in both cases.)
- **Standards laboratory**. Two components are taken into account: the difference between the possible value of this factor from 1.000 (0.1% for the M30013 and 0.2% for the Markus), and the standard deviation associated with the measurement in a C60o beam. Both will yield a good estimate of the uncertainty value. However, the latter component is caused by the reproducibility of measurements with the C60o machine, which is usually assumed to be much better than the measurements achieved with an accelerator (<<0.1%). Hence, this component can be considered negligible.

When both components are combined in quadrature, they reach an uncertainty of 0.14% and 0.25% for the M30013 and Markus chambers respectively (Table 4).

**Table 4:** Estimated relative standard uncertainty of the polarity correction factor and of its components, for two chambers types: a cylindrical PTW 30013 (PTW, Freiburg, Germany) and a plane‐parallel Markus chamber (PTW)

Component		Photons, M30013			Electrons, Markus		
		Uncertainty type A (%)		Uncertainty type B (%)	Uncertainty type A (%)		Uncertainty type B (%)
User	Reproducibility	0.1			0.15		
Standards laboratory	Non‐applied correction			0.1			0.2
**Combined uncertainty** (k=1)			0.14			0.25	

#### A.8 Ion recombination ${\left(k_{S}\right)}_{Q}/{\left(k_{S}\right)}_{Q_{0}}$

##### A.8.1 Values

- **User**. Because pulsed beams are used, the ion recombination factor is calculated using a quadratic fit of the two‐voltage method (TRS‐398, p. 50). This calculation procedure has been considered to be appropriate once the linear dependence of 1/M and 1/V required by the Boag theory is determined to be satisfied. Some publications show that this linear dependence is not valid over a certain threshold voltage for various chambers, both plane‐parallel^(^ ^15^ ^,^ ^20^ ^–^ ^23^ ^)^ and cylindrical.^(^ ^24^ ^,^ ^25^ ^)^
- **Standards laboratory**. The ion recombination is provided by instrument specifications according to the rate in the presence of continuous radiation (C60o). Taking into account the dose rate used in the standards laboratory, 0.1 Gy/min, the ion recombination is negligible, and therefore a value of 1.000 is assigned to this factor. In the case of the Markus chamber, these values are confirmed in the literature.^(^ ^15^ ^,^ ^20^ ^)^

##### A.8.2 Uncertainties

- **User**. The following components have been evaluated to estimate the uncertainty:
*Two‐voltage method*. The two‐voltage uncertainty assignment is based on the difference between the expected value from the Boag theory and the value measured by the two‐voltage method. The differences found were under 0.03% for photons and 0.11% for electrons.
*Polynomial fit error*. The maximum deviation found in data provided by Weinhous and Meli^(26)^ is 0.1%. The uncertainty of this component is therefore estimated at 0.06%.
*Error attributable to the parameters* $V_{1}/V_{2}$ and $M_{1}/M_{2}$. A function
  (6) $$k_{\;\;S}^{*}=k_{S}∙f(\alpha ,\beta ),$$
  in which $\alpha =V_{1}/V_{2}\;\text{and}\;\beta =M_{1}/M_{2}$, is used to estimate this component. For well‐defined values of α and β, *f*(α,β) takes the value 1.
  Using the combined uncertainty formula
  (7) $$\frac{u^{2}(k_{S}^{*})}{{\left(k_{S}^{*}\right)}^{2}}=\frac{u^{2}(k_{S})}{{\left(k_{S}\right)}^{2}}+{\left(\frac{1}{k_{S}^{*}}\right)}^{2}\left[{\left(\frac{\partial k_{S}^{*}}{\partial \alpha }\right)}^{2}u^{2}(\alpha )+{\left(\frac{\partial k_{S}^{*}}{\partial \beta }\right)}^{2}u^{2}(\beta )\right],$$
  in which the first term is the relative variance associated with the uncertainties of the two‐voltage method and the polynomial fit, finding the other two addends requires calculation of the variations of the ion recombination factor with the possible errors in the set of $V_{1}/V_{2}$ (Δα: electrometer resolution when selecting the two voltages) and in the measurements $M_{1}/M_{2}\;(\Delta \beta )$. Quantifying the increasing expressions $\Delta k_{S}/\Delta \alpha$ and $\Delta k_{S}/\Delta \beta$ is therefore necessary. The polynomial fit of the two‐voltage method can be used for this purpose. This component is estimated to be less than 0.03%.
- **Standards laboratory**. The two‐voltage method is also used to quantify the ionic recombination in the presence of continuous radiation. In this case, the difference between the value obtained with this method and the value obtained with the model provided by Zankowski and Podgorsak^(27)^ reaches maximum values of 0.16% for cylindrical chambers and 0.28% for plane‐parallel chambers.^(28)^ The Zankowski model takes into account initial recombination, ionic diffusion, and charge multiplication. This deviation is fully assigned as relative standard uncertainty. The uncertainty introduced by the influences of $V_{1}/V_{2}$ and $M_{1}/M_{2}$ is again estimated within 0.03%. (As mentioned earlier, the reproducibility for a C60o machine is better than that for an accelerator, which could mean a smaller value for $\Delta k_{S}/\Delta \beta$.)

Previous models do not take into account the effect of the free electron collection,^(^ ^29^ ^,^ ^30^ ^)^ and so an adequate uncertainty should be assigned to account for this lack of correction. For the Markus chamber, the increased uncertainty that results from not considering this effect can be calculated by model 1(a) as proposed by Boag et al.,^(30)^ obtaining values of approximately 0.05%. The error for the cylindrical chamber reaches 0.1%. In continuous radiation and considering the calibration dose rate, the effect is negligible.^(29)^

The combined uncertainty deduced from the quadratic sum of these components reaches values of 0.16% and 0.21% for the M30013 and the Markus chamber respectively (Table 5).

**Table 5:** Estimated relative standard uncertainty of the recombination correction factor,^a^ for two chambers types: a cylindrical PTW 30013 (PTW, Freiburg, Germany) and a plane‐parallel Markus chamber (PTW)

Component		Uncertainty (%)	
		Photons, M30013	Electrons, Markus
User	Two‐voltage method	0.03	0.11
	Polynomical fit	0.06	0.06
	α, β^b^	0.03	0.03
	Free electrons	0.10	0.05
Standards laboratory	Zankowski model	0.09	0.16
	α,β^b^	0.03	0.03
**Combined uncertainty** (k=1)		**0.16**	**0.21**

a All values arise from type B uncertainty analysis.

b $\alpha =V_{1}/V_{2}$, and $\beta =M_{1}/M_{2}$.

Summarizing, the relative combined standard uncertainty of the corrected reading is 0.49% for photons measured with the PTW 30013 chamber and 0.70% for electrons measured with the Markus chamber (Table 6). An analog study of the NE 2571 chamber in photon beams obtained a relative uncertainty of 0.44%.

**Table 6:** Estimated relative standard uncertainties of the corrected reading and of its various components

Component	Uncertainty (%)	
	Photons, M30013	Electrons, Markus
Reading	0.29	0.48
Beam monitor	0.14	0.14
Pressure	0.09	0.09
Temperature	0.18	0.18
Humidity	0.17	0.17
Electrometer calibration	0.14	0.14
Polarity	0.14	0.25
Recombination	0.16	0.21
**Combined uncertainty** (k=1)	**0.49**	**0.70**

### B. Calibration factor $N_{D,w,Q_{0}}$

The calibration factors for both PTW chambers were supplied by the SSDL PTW, traceable to PTB, with a relative standard uncertainty of 1.1%. This value is slightly higher as compared with other laboratories. For example, Ciemat, the SSDL in Spain, which is traceable to BIPM, disseminates the absorbed dose to water with an uncertainty of approximately 0.5%. Two reasons explain this higher value:

- First, the PTB primary standard was based on the Fricke chemical method, which leads to an approximated uncertainty of 0.7%. The major contribution to this value (approximately 0.5%) is attributable to the original calibration of the Fricke solution through total absorption of a 5.6 MeV electron beam and the later transference to the C60o photon beam. An additional contribution of approximately 0.2% comes from a supposition of the independence of the radiation type and energy and of the radiation chemical yield in the experimental solution. The difference with the BIPM ionometric standard should be noted: The associated uncertainty is approximately 0.4%.
- Second, the dissemination to the user's chambers from the PTW laboratory demands an additional, higher‐than‐usual contribution to the uncertainty. It should not involve any appreciable increase of the final uncertainty. At Ciemat, the NE chamber was calibrated with an uncertainty of 0.55%, which means that the uncertainty rises from 0.4% (BIPM) to 0.55%. In the case of PTW, the increase is higher: from 0.7% (PTB) to 1.1%.

### C. Beam quality correction factor $k_{Q,Q_{0}}$

The factor $k_{Q,Q_{0}}$ corrects for the effects of the difference between the user beam quality *Q* and the reference beam quality $Q_{0}$. Ideally, this factor should be experimentally evaluated by the laboratory for each chamber according to the user beam quality, but only a few PSDLs in the world can perform such experiments. This method would offer the advantage of distinguishing the individual energy response of each chamber. However, theoretical calculation is the common practice for determination of $k_{Q,Q_{0}}$. Therefore chamber‐to‐chamber variations in response are ignored, and the related uncertainty is larger than for experimentally determined values.

In Bragg–Gray conditions, the expression $k_{Q,Q_{0}}$ is the relationship between the water—air stopping power ratio, the mean energy expended in air per ion pair formed, and the various perturbation factors of the chamber, both in the user beam quality and in the reference beam quality. The data for these parameters can be found in Appendix B of TRS‐398, and they are supposed to be the best choice in the current state of the art for ionization chamber dosimetry. The uncertainty value given by TRS‐398 for this calculated factor is 0.9% for photon beams measured with a cylindrical chamber and 1.7% for electron beams measured with a plane‐parallel chamber. When experimental values for $k_{Q,Q_{0}}$ are used, the standard uncertainty is placed at 0.7% and 0.8% respectively. In the case of plane‐parallel chambers calibrated in a C60o beam, the large uncertainty associated with the calculated $k_{Q,Q_{0}}$, 1.7%, comes mainly from the factor $p_{\text{wall}}$, because of variations of up to 3% found between chambers of the same type. In a recent publication, Christ et al.^(31)^ concluded that chamber‐to‐chamber variations for various plane‐parallel chambers (Roos, Markus, and Advanced Markus) are less than 0.3%. In addition, other recent papers by Palm et al.^(32)^ and Stewart and Seuntjens^(33)^ have verified this number; they present consistent $p_{\text{wall}}$ values for various chamber types. The relative standard uncertainty for this factor is estimated at 0.5%.^(31)^ The new combined uncertainty in the calculated $k_{Q,Q_{0}}$ for electron beams when the reference quality $Q_{0}$ is C60o can be estimated at 1.0%. This value has been used in the present work to carry out the estimates of uncertainty.

In TRS‐398, the values for $k_{Q,Q_{0}}$ are presented as a function of ${\text{TPR}}_{20,10}$ in the case of photons and $R_{50}$ in the case of electrons. These are the parameters that specify the beam quality. In addition, an extra component will be present: the uncertainty in the interpolation of the tables in TRS‐398 to deduce the factor $k_{Q,Q_{0}}$. It is first necessary to evaluate the uncertainty in the determination of ${\text{TPR}}_{20,10}$. That value is near 0.4%. The largest component is related to the use of ionization ratios instead of ratios of absorbed dose in the determination of ${\text{TPR}}_{20,10}$. That approximation is a good one, within 0.3%,^(34)^ because of the slow variation in depth of water– air stopping power ratios and the non‐dependence of perturbation factors with depths beyond the dose maximum. When the interpolation is made, the deviation in $k_{Q,Q_{0}}$ will be given by the range of variation in ${\text{TPR}}_{20,10}$ measurement (±0.4%), which is 0.1% at a maximum. The same procedure is applied to the electron beams. The uncertainty estimated for them is less than 0.05%. The values of these components are negligible as compared with the uncertainty associated with the calculation of $k_{Q,Q_{0}}$.

### D. Reference conditions

The possibility of inaccuracy when beam calibration conditions are established, either in chamber positioning at the source‐to‐surface distance (SSD), in the depth (*z*), or in the setting of the field size (*L*), requires evaluation of the possible magnitude of such deviation and assignment of an appropriate uncertainty. To quantify the value of that uncertainty, the following function is used:

(8) $$D_{\;\;w,u}^{*}=D_{w,u}∙f(d_{\text{SSD}},d_{L},d_{z}),$$

where *f* is equal to 1 in well‐defined and typified measurement conditions. The combined uncertainty is represented by the expression

(9) $$\frac{u^{2}(D_{w,u}^{*})}{{\left(D_{w,u}^{*}\right)}^{2}}=\frac{u^{2}(D_{w,u})}{{\left(D_{w,u}\right)}^{2}}+{\left(\frac{1}{D_{w,u}^{*}}\right)}^{2}\left[{\left(\frac{\partial D_{w,u}^{*}}{\partial d_{SSD}}\right)}^{2}u^{2}(d_{SSD})+{\left(\frac{\partial D_{w,u}^{*}}{\partial d_{L}}\right)}^{2}u^{2}(d_{L})+{\left(\frac{\partial D_{w,u}^{*}}{\partial d_{z}}\right)}^{2}u^{2}(d_{z})\right],$$

in which the first addend is the relative variance corresponding to the absorbed dose to water under reference conditions, and the other addends are the relative variances related to the three variables quoted.

First, an uncertainty must be associated with each parameter:

- **SSD**. The maximum deviation in optical distance indicator, less than 1 mm, is taken. This value is provided by the geometric controls periodically made. In addition, a visual error of thickness in the line of the optical distance indicator is added. Considering these two factors, a maximum possible error $\left(d_{\text{SSD}}\right)$ in the SSD setting of 1 mm is assumed, which means, associating a uniform distribution, an uncertainty value of $u(d_{\text{SSD}})=0.06$ cm.
- **Field size**. According to the geometric controls, the maximum deviation in the light field size is 1 mm. On the other hand, the maximum deviation between the radiation field size and the light field size is around 1 mm. Assuming a maximum possible error $\left(d_{L}\right)$ of 2 mm, the uncertainty is estimated to be $u(d_{L})=0.12$ cm.
- **Depth**. The possible error comes from the visual error when positioning the chamber and from the error of the movement array of the beam analyzer. Another source of uncertainty is the non‐applied correction attributable to water density. For a typical measurement temperature of 20 °C, the water density amounts to approximately $0.9982\;g/{\text{cm}}^{3}$. When the chamber is “placed” at the reference depth, the real position is some tenths of millimeters deeper (approximately 0.2 mm for photons and less than 0.1 mm for electrons at a temperature of 20 °C). In addition, an extra component has been considered in the case of electron beams, because the reference depth is specified in terms of $R_{50}$ in TRS‐398. Considering all these components, the estimated uncertainty is $u(d_{z})=0.07$ cm for photons and 0.06 cm for electrons.

Second, the rate of relative dose variation should be known, considering the possible setting error of any of the previous parameters—that is, [(ΔD/D)/Δd]. Experimental measurements were taken to estimate each of these incremental expressions. Table 7 presents the results. The combined uncertainty is calculated using the second part of expression 9.

**Table 7:** Relative dose variation according to the possible error in the experimental setup of source‐to‐surface distance (SSD), field size (L), and measurement depth (z)^a^

	Saturn 43							
Nominal energy	X6 MV	X25 MV	e6 MeV	e9 MeV	e12 MeV	e15 MeV	e18 MeV	e21 MeV
$\left(\Delta D_{w.u}/D_{w,u})/\Delta d_{\text{SSD}}({\text{cm}}^{-1}\right)$	0.019	0.019	0.022	0.022	0.021	0.021	0.021	0.021
$\left(\Delta D_{w.u}/D_{w,u})/\Delta d_{L}({\text{cm}}^{-1}\right)$	0.025	0.025	0.035	0.035	0.035	0.035	0.035	0.035
$\left(\Delta D_{w.u}/D_{w,u})/\Delta d_{z}({\text{cm}}^{-1}\right)$	0.049	0.074	0.101	0.061	0.034	0.027	0.034	0.042
**Combined uncertainty (%)**	**0.47**	**0.62**	**0.72**	**0.55**	**0.47**	**0.45**	**0.47**	**0.49**

a The combined uncertainty is obtained using equation 9 (see text). The results obtained for the Saturn 40 are very similar.

Table 7 shows that the most relevant component is the setting of the chamber at the reference depth. Chamber positioning is more critical for low‐energy electrons because of the sudden decrease in the dose with depth at this range. The position of the chamber at the reference depth $\left(z_{\text{ref}}\right)$ is near the peak of the depth‐dose curve, which is very narrow for low energies. However, flattening of the curve in the peak occurs with increasing energies. For medium energies, $z_{\text{ref}}$ is at the maximum, so that the reading is relatively insensitive to chamber position. At high energies, $z_{\text{ref}}$ moves beyond the maximum, and so the dose gradient can become significant, with uncertainty again increasing.

When all the components evaluated to this point are combined in quadrature, the relative standard uncertainty in the determination of the absorbed dose to water under reference conditions is obtained (Table 8):

- 1.6% for photons measured with the M30013 cylindrical chamber,
- 1.3% for photons measured with the NE 2571 cylindrical chamber, and
- 1.7% for electron beams measured with the Markus plane‐parallel chamber.

**Table 8:** Estimated relative standard uncertainty of the absorbed dose to water under reference conditions using the $N_{D,w}$ formalism and of its components^a^

Component	Cylindrical chamber M30013 X6 – X25 MV	Cylindrical chamber NE 2571 X6 – X25 MV	Plane‐parallel chamber Markus e15 – e6 MeV
Corrected reading	0.49	0.44	0.70
Calibration factor $\left(N_{D,w}\right)$	1.1	0.55	1.1
Beam quality correction factor $\left(k_{Q,Q0}\right)$	0.9	0.9	1.0
Reference conditions	0.47 0.62	0.47 0.62	0.45 0.72
**Combined uncertainty** (k=1)	**1.6**	**1.3**	**1.7 1.8**

a Based on a chamber calibration in C60o beam radiation. The electron energies shown are the limits of variation in the uncertainty value for the reference conditions. For the rest of the energies, the uncertainty is found to be between such two values.

Comparing the results for photon beams, it can be seen that the uncertainty changes from 1.6% to 1.3% if the chamber calibrated at Ciemat is used. The reason is that the calibration factor component is significantly smaller (0.55% as compared with 1.1% for the PTW 30013). This change will also occur for electron beams if a typical uncertainty 0.6% in the calibration factor is considered: the 1.7% uncertainty would be reduced to 1.5%.

## IV. DISCUSSION

First, as can be seen from the present work, the evaluation of uncertainties is not an easy task because it is partly based on subjective considerations (type B uncertainties). The purpose of the detailed study here is to include most of the uncertainty components in the determination of the absorbed dose to water in high‐energy beams for external radiotherapy. The estimations presented are specific and applicable to the equipment used at our institution. However, our work can be used as model for other medical physicists, so that they can evaluate their own uncertainty in the process of calibration. It also provides an idea about which components are the most relevant and which components contribute insignificantly and therefore do not need to be evaluated. On the other hand, whenever modern equipment is used, the uncertainty values obtained will probably not vary excessively in comparison with those documented by other users.

Other publications have outlined how to conduct an uncertainty analysis for beam calibration, but those publications are poorly documented, in that the values presented have an unknown origin. In the present paper, we explain and justify all uncertainty values.

The components associated with the calibration factor have been shown to be able to significantly change the uncertainty in the absorbed dose to water. When chambers calibrated at PTW are considered, the contribution of $N_{D,w}$ becomes the major component.

The other important contribution to the final uncertainty is the beam quality correction factor $k_{Q,Q_{0}}$. The explanation comes from evaluation of the correction factor by a theoretical calculation. Thus, the response of each individual chamber is not taken into account. In future, all standards laboratories are expected to start directly measuring the factor $k_{Q,Q_{0}}$ in the user‐quality beam. Therefore, chamber‐to‐chamber variations will be considered, and a reduction in the $k_{Q,Q_{0}}$ uncertainty to 0.7% from 0.9% (photon beams) and to 0.8% from 1.7% (electron beams) will be expected. With these values, the uncertainty in the determination of absorbed dose to water is estimated to become 1.1% for photons and 1.4% for electrons.

For electron beams, TRS‐398 advises using a cross‐calibration to calibrate plane‐parallel chambers to avoid the problems associated with $p_{\text{wall}}$ and its large uncertainty. However, some recent publications were shown to include values less than that proposed by TRS‐398. In addition, it should be noted that, using the cross‐calibration procedure, the component associated with $N_{D,w,Q_{cross}}^{pp}$ would increase considerably. A cross‐calibration of the Markus plane‐parallel chamber against the M30013 cylindrical chamber was recently conducted at our center. In that case, the expression to determine dose is given by TRS‐398 (p. 85). An analysis of the components of that expression, similar to the analysis here, puts the uncertainty at 1.7%−1.8% depending on the energy, the same value as was obtained with chamber calibration in C60o beam quality.

On the other hand, adoption of the formalism based on the absorbed dose to water (as compared with the formalism based on air kerma) should entail an uncertainty reduction in this part of the process. An estimate of the uncertainty in the case of the $N_{D,\text{air}}-N_{k}$ formalism has been made. First, because of the similar measurement conditions, the uncertainties associated with the corrected reading and with the reference conditions are estimated to be equal to those obtained with the procedure based on the absorbed dose to water. Another source of uncertainty is the determination of the chamber factor $N_{D,\text{air}}$ based on an $N_{K}$ calibration. The uncertainty value provided by Ciemat in $N_{K}$ for the NE 2571 chamber is 0.45%. The remaining parameters that enter into the determination of $N_{D,\text{air}}$ and of the components associated with the perturbation factors in the user beam $p_{Q}$, and to the water–air stopping power ratio $s_{w,\text{air}}$, were taken from Huq and Andreo.^(7)^ Thus the uncertainty in $N_{D,\text{air}}$ can be set for a cylindrical chamber at 0.8%. A reasonable estimate of the cross‐calibrated plane‐parallel chamber uncertainty in $N_{D,\text{air}}$ may be 1.3%. The uncertainty of the perturbation factors $p_{Q}$ and $s_{w,\text{air}}$ is 0.9% for photons measured with a cylindrical chamber and 0.7% for electrons measured with a cross‐calibrated plane‐parallel chamber. Previous components are combined in quadrature to obtain uncertainty values of 1.4% and 1.7% – 1.8% respectively (Table 9).

**Table 9:** Estimated relative standard uncertainty of the absorbed dose to water under reference conditions using the $N_{D,\text{air}}$ formalism and of its components^a^

Component	Cylindrical chamber X6 – X25 MV	Plane‐parallel chamber e15 – e6 MeV
Corrected reading	0.44	0.70
Calibration factor $\left(N_{D,\text{air}}\right)$	0.8	1.3
Perturbation factors $\left(p_{Q}/s_{w,\text{air}}\right)$	0.9	0.7
References conditions	0.47 0.62	0.45 0.72
**Combined uncertainty** k=1	**1.4**	**1.7 1.8**

a The values shown for electron beams are based on a plane‐parallel chamber cross‐calibrated in a high‐energy electron beam.

The comparison between the uncertainty values obtained by formalisms based on absorbed dose to water and air kerma (if a typical uncertainty of 0.6% for the calibration factor $N_{D,w}$ is assumed) results in 1.3% and 1.4% respectively for photon beams and in 1.5% and 1.7% respectively for electron beams measured with a cross‐calibrated plane‐parallel chamber. These findings make it evident that the estimated uncertainty is similar in both cases.

## V. CONCLUSIONS

Results show a typical uncertainty in the determination of absorbed dose to water during beam calibration of approximately 1.3% for photon beams and 1.5% for electron beams (k=1 in both cases) when the $N_{D,w}$ formalism is used and $k_{Q,Q_{0}}$ is calculated theoretically. Similar values are found when the $N_{D,\text{air}}$ formalism is used, and so we question the argument of an uncertainty reduction in favor of the method based on $N_{D,w}$. At first, the total application of the $N_{D,w}$ formalism is based on specific calibrations of each chamber in the user beam quality, and so differences between chambers of the same type would be considered and consequently uncertainty would be reduced. Considering this fact, a possible uncertainty of 0.7% in photon beams and 0.8% in electron beams for the factor $k_{Q,Q_{0}}$ can be assumed. The uncertainty in this step of the process could therefore be placed at values near 1.1% for photon beams measured with a cylindrical chamber and 1.4% for electron beams measured with a plane‐parallel chamber. The global impact at the clinical level caused by this uncertainty reduction can be a question for future research.
